# Retinitis Pigmentosa: Genes and Disease Mechanisms

**DOI:** 10.2174/138920211795860107

**Published:** 2011-06

**Authors:** Stefano Ferrari, Enzo Di Iorio, Vanessa Barbaro, Diego Ponzin, Francesco S Sorrentino, Francesco Parmeggiani

**Affiliations:** 1 The Veneto Eye Bank Foundation, Mestre-Venice, Italy; 2 Department of Ophthalmology, University of Ferrara, Ferrara, Italy

**Keywords:** Syndromic retinitis pigmentosa, non-syndromic retinitis pigmentosa, retina, dominant, recessive, X-linked, mutations.

## Abstract

Retinitis pigmentosa (RP) is a group of inherited disorders affecting 1 in 3000-7000 people and characterized by abnormalities of the photoreceptors (rods and cones) or the retinal pigment epithelium of the retina which lead to progressive visual loss. RP can be inherited in an autosomal dominant, autosomal recessive or X-linked manner. While usually limited to the eye, RP may also occur as part of a syndrome as in the Usher syndrome and Bardet-Biedl syndrome. Over 40 genes have been associated with RP so far, with the majority of them expressed in either the photoreceptors or the retinal pigment epithelium. The tremendous heterogeneity of the disease makes the genetics of RP complicated, thus rendering genotype-phenotype correlations not fully applicable yet. In addition to the multiplicity of mutations, in fact, different mutations in the same gene may cause different diseases. We will here review which genes are involved in the genesis of RP and how mutations can lead to retinal degeneration. In the future, a more thorough analysis of genetic and clinical data together with a better understanding of the genotype-phenotype correlation might allow to reveal important information with respect to the likelihood of disease development and choices of therapy.

## 1. WHAT IS RETINITIS PIGMENTOSA?

Retinitis pigmentosa (RP) is a class of diseases involving progressive degeneration of the retina, typically starting in the mid-periphery and advancing toward the macula and fovea. Typical symptoms include night blindness followed by decreasing visual fields, leading to tunnel vision and eventually legal blindness or, in many cases, complete blindness [1]. On the cellular level, this correlates with a predominantly affected rod photoreceptor system. In later stages, the disease may further affect the cone photoreceptor eventually causing complete blindness. The diseased photoreceptors undergo apoptosis [2], which is reflected in reduced outer nuclear layer thickness within the retina, as well as in lesions and/or retinal pigment deposits in the fundus. Patients may lose a significant portion of their photoreceptors before experiencing loss of visual acuity. Clinical hallmarks are (i) an abnormal fundus with bone-spicule deposits and attenuated retinal vessels, (ii) abnormal, diminished or absent a- and b-waves in the electroretinogram (ERG) and (iii) reduced visual field. Symptoms typically start in the early teenage years and severe visual impairment occurs by ages 40 to 50 years (reviewed in [3]). A multicenter study evaluating visual acuity (VA) data from 999 patients (age 45 years or older) with different genetic subtypes of RP found that 52% had a VA of 20/40 or better, 69% of 20/70 or better, 25% of 20/200 or worse and 0,5% had no light perception in both eyes [4]. There are also early-onset forms of RP (the earliest is indistinguishable from Leber congenital amaurosis) and other late-onset or even non-penetrant forms.

RP, broadly defined to include simple, syndromic and systemic disease, has a worldwide prevalence of 1:3000 to 1:7000 people. Usually, no sex predilection exists. However, as X-linked RP is expressed only in males, statistically men may be affected slightly more than women. RP shows no ethnic specificity, but RP caused by mutations in particular genes may be more frequent in certain isolated or consanguineous populations (such as the USH3 gene associated with type III Usher syndrome, normally rare, but more common among Finns and Ashkenazi Jews).

The genetics of RP is varied. After the first report describing a linkage of an RP locus to a DNA marker on human chromosome X in 1984 [5], over 40 genes have been associated with RP (Table**1**). Non-syndromic or “simple” cases may be inherited as an autosomal dominant (20-25%); Fig. (**1**)), autosomal recessive (15-20%; Fig. (**2**)), X-linked recessive (10-15%; Fig. (**3**)), or sporadic/simplex traits (30%), and 5% may be early-onset and grouped as part of Leber congenital amaurosis (LCA). Rarer forms also exist, such as X-linked dominant, mitochondrial, and digenic (due to mutations in two different genes). While RP is a disease usually limited to the eye, there are syndromic forms involving multiple organs and pleiotropic effects as well as systemic forms wherein the retinal disease is secondary to a system-wide pathology. The most frequent forms of syndromic RP are Usher syndrome (RP usually develops by the early teenage years) and Bardet-Biedl syndrome.

Approximately 20-30% of patients with RP have an associated non-ocular disease and would be classified as having syndromic RP. A list of non-syndromic and syndromic RP is maintained through RetNet (http://www.sph.uth.tmc.edu/retnet/). Table **2** shows the overall prevalence of non-syndromic or “simple” RP (not affecting other organs or tissues) and the proportion of the most common syndromic (affecting other systems such as hearing) or systemic (affecting multiple organs) RP.

The genetics of RP is complicated and clear genotype-phenotype correlations are not yet possible. In addition to the multiplicity of mutations, in fact, (i) different mutations in the same gene may cause different diseases and (ii) the same mutation can exhibit intra- and inter-familial phenotypic variability (as for BEST1 mutations). For example, the vast majority of *RHO* mutations show a classical autosomal dominant inheritance leading to RP. Nevertheless, a few mutations show an autosomal recessive inheritance or lead to night blindness only [6]. Similarly, *RPGR*, the major X-linked recessive RP gene, is associated with mutations in male patients and no male-to-male transmission of the phenotype has ever been observed. However, families with dominant inheritance pattern and female carriers showing disease symptoms of variable degree (likely due to the dominant nature of some of the mutations or non-random X-inactivation in the affected tissue) have also been described [6]. Furthermore, mutations in some genes are associated with incomplete penetrance, thus making genotype-phenotype correlations even more difficult.

In the next paragraphs we will overview the genes involved in the genesis of RP and how mutations can lead to patho-physiological consequences. A homogeneous and unique pattern of disease development cannot be envisaged as genes affected in RP are multiple and cover different steps of the visual cycle (phototransduction, retinal metabolism, tissue development and maintenance, cellular structure, splicing). For each of the main genes involved in RP, we will therefore evaluate the frequency of the mutations associated and how these lead to retinal degeneration and eventually RP.

## 2. THE VISUAL CYCLE

In order to understand better how mutations in the genes involved in RP affect the activity of the proteins, a brief outline of the molecular bases of vision needs to be described.

Human vision begins when light enters the eye and is focused by the lens onto the photosensitive tissue at the back of the eye, the retina (Fig. (**4**)). Light-sensitive cells in the retina - the rod and cone photoreceptors – capture the incoming photons. The photoreceptors are polarized cells that consist of a synaptic region, a cell body, an inner segment (IS) and an outer segment (OS). The “molecular machinery” involved in biosynthesis, energy metabolism and membrane trafficking reside within the IS, which is connected to the OS *via *the so called connecting cilium. The OS is comprised of membranous discs surrounded by a plasma membrane. Over 90% of the total protein in the OS is rhodopsin, which resides both within the membrane of the discs and in the surrounding plasma membrane. Rhodopsin is one of the most extensively studied G-protein coupled receptors and its ligand-binding domain is buried within the transmembrane portion of the molecule. It is highly conserved among vertebrates and rhodopsin-like proteins can also be found in lower vertebrates. Vision in vertebrates begins with the absorption of light by the rod photoreceptor “visual pigment”, rhodopsin, which consists of an apoprotein, opsin (a single 384 amino acid polypeptide chain), and a chromophore (11-cis retinaldehyde, derived from vitamin A) attached to the opsin by a Schiff base bond. The absorption of light by rhodopsin causes isomerization of 11-*cis* retinal to *all-trans* retinal, which changes opsin conformation, thus initiating vision. *All-trans* retinal is liberated from opsin in the photoreceptor cells and is reduced to *all-trans* retinol, which moves to the retinal pigment epithelium (RPE). For vision to continue, 11-*cis* retinal has to be produced again from *all-trans* retinol in the RPE. This process is carried out by a 65kDa isomerohydrolase expressed in the RPE called RPE65 [7, 8]. The light-activated rhodopsin interacts with and activates its cognate G (GTP-binding) protein, transducin, which subsequently initiates a canonical second-messenger signaling pathway *via *the heterotetrameric phosphodiesterase (PDE) 6 complex.

## 3. NON-SYNDROMIC RETINITIS PIGMENTOSA

### 3.1. Autosomal Dominant RP

To date mutations in 22 different genes are associated with autosomal dominant RP (adRP) (http://www.sph.uth. tmc.edu/retnet/; Fig. (**1**) and Table **3**). However, only a few of these account for a relevant percentage of cases and include RHO (26,5%), PRPH2/RDS (5-9,5%), PRPF31 (8%) and RP1 (3,5%).

#### Rhodopsin

As above described, rhodopsin (RHO) is the first component of the visual transduction pathway and is activated by absorption of light in the rod photoreceptor cells of the retina (reviewed in [10]). Mutations in the *RHO* gene account for 30 to 40% of all adRP, with more than 100 different mutations (but Pro23His is found in approximately 10% of Americans affected with adRP). The analysis of mutant rhodopsins suggests that mutations can impair protein folding, 11-*cis* retinal chromophore binding, G-protein coupling/activation, and/or cellular trafficking of the rhodopsin protein. In addition, mutations in RHO can also lead to autosomal dominant congenital night blindness (CNB) and autosomal recessive retinitis pigmentosa (arRP). A documented case of arRP resulted from a mutation in the RHO gene that led to a protein that was missing the 6^th^ and 7^th^ transmembrane domains, including the 11-cis retinal binding site [11]. Differently, RHO mutants associated with CNB appear to arise as a result of a constitutively active form of opsin that can catalytically activate the G-protein transducin in the absence of chromophore and in absence of light. Despite the amount of knowledge in adRP caused by mutations in RHO, therapeutic approaches have not proceeded at the same pace. Recently, visual function in P23H transgenic rats was restored following subretinal delivery of recombinant AAV vectors expressing the Bip/Grp78 gene (a chaperone protein able to promote trafficking of misfolded Pro23His rhodopsin to the cell membrane) [12]. Improved retinal function was also obtained in a mouse model of dominant RP following subretinal administration of recombinant AAV vectors carrying RNAi-based suppressors [13].

#### Pre-mRNA Processing Factor 31 (PRPF31)

PRPF31 was recently suggested to play a major role in adRP, with mutations ranging from 1 to 8% in adRP cohorts from various geographical origins (6,7% in a French adRP cohort) and higher frequencies in the United States [14]. PRPF31 is a pre-mRNA splicing factor of 61kDa, which is integral to the U4/U6+U5 trimer. To date over 40 mutations have been located in different parts of the gene and comprise missense substitutions, splice-site mutations, deletions and insertion. Interestingly, PRPF31 is one of three pre-mRNA splicing factors identified as causing adRP. Two other pre-mRNA splicing factors have also been implicated in adRP: PRPF3 (RP18) found in 1% of cases and PRPF8 (RP13) found in 3% of cases (Table**3**).  Proteins encoded by these genes are essential for splicing in all cell types, yet the pathologic effects of mutations in all 3 genes is seen only in rod photoreceptors. Incomplete penetrance is one of the unique features associated with mutations in *PRPF31*. Asymptomatic carriers can have affected parents and children, which can significantly complicate determining the mode of inheritance, thereby hindering the genetic counseling of the family. Previously, it has been reported that symptomatic individuals experience night blindness and loss of visual field in their teens and are typically registered as blind when they reach their 30s. Detailed haplotype analysis in PRPF31-linked families indicated that asymptomatic patients inherit a different wild-type allele from the one inherited by their symptomatic siblings, suggesting the existence of differentially expressed wild-type alleles that can potentially determine the penetrance of the disease symptoms. The high expression level of the wild-type allele may compensate for the nonfunctional mutant allele, whereas the low-expressing wild-type allele is inadequate to reach the required photoreceptor-specific PRPF31 activity threshold [15, 16].

#### Peripherin 2

The peripherin 2 gene (*PRPH2*), formerly known as the retinal degeneration slow gene (*RDS*), consists of 3 exons and encodes a 39-kDa integral membrane glycoprotein with 346 amino acids. The protein includes 4 transmembrane domains (M1-M4) and a large intradiscal domain (known as the D_2_ loop) and is located at the outer segment discs of the rod and cone photoreceptors. It forms a homo-oligomeric structure with itself and a mixture of homotetrameric and heterotetrameric complexes with another membrane protein (ROM1) [17]. This structure is important in disc morphogenesis and stabilization. Mutations in *PRPH2* that cause RP were first described in 1991 and account for 5-9,5% of adRP cases. Interestingly, cases of digenic RP have been observed following the simultaneous presence of a mutation in the PRPH2 (RDS) gene and a mutation in the ROM1 gene [18]. In all cases reported, the same PRPH2 (RDS) mutation (L185P) was found, although three different ROM1 mutations were identified in these families. Studies carried out in Prph2Rd2/Rd2 mice have shown that the genetic defect can be corrected both morphologically and functionally by *in vivo* gene transfer. The ERGs of Prph2Rd2/Rd2 mice have greatly diminished a-wave and b-wave amplitudes, which decline to virtually undetectable concentrations by two months. Subretinal injection of recombinant adeno-associated virus (AAV) encoding a Prph2 transgene resulted in stable generation of outer segment structures and formation of new stacks of discs containing both perpherin-2 and rhodopsin, which in many cases were morphologically similar to normal outer segments. Moreover, the re-establishment of the structural integrity of the photoreceptor layer also resulted in electrophysiological correction [19].

#### Retinitis Pigmentosa 1

The Retinitis Pigmentosa 1 (*RP1*) locus was originally mapped by linkage testing in a large adRP family in southeastern Kentucky and the RP1 gene subsequently identified by positional cloning. Mutations in *RP1* cause both dominant and recessive forms of RP. Mutations in the RP1 gene account for approximately 5-10% of all adRP (4% in the United States) with the Arg677X mutation accounting for ½ of the total [from http://www.sph.uth.tmc.edu/retnet/]. The RP1 gene encodes a 240-kD retinal photoreceptor-specific protein. RP1 is expressed prominently, if not only, in the photoreceptor cells of the retina and is involved in the correct orientation and higher order stacking of outer segment discs. RP1 is a microtubule-associated protein forming part of the photoreceptor axoneme and thus plays an important role in photoreceptor function [20, 21]. Clinical characterization of patients with *RP1* disease shows notable differences in visual field diameters and ERG amplitudes in patients of the same age who share the same primary mutations. In general, variation in the phenotypes of genetic disorders can be attributed to allelic heterogeneity, environmental factors, and genetic modifiers. For *RP1* disease, genetic modifiers are thought to be especially important because variation in the severity of disease is observed in patients with the same primary mutation and because environmental exposures are likely to be relatively similar within families.

#### Diagnosis of adRP

As for the molecular diagnosis of adRP, an array has recently been developed and shown to enable the analysis of 385 mutations in 16 different genes involved in adRP: CA4, FSCN2, IMPDH1, NRL, PRPF3, PRPF31, PRPF8, RDS, RHO, ROM1, RP1, RP9, CRX, TOPORS, and PNR (www.asperbio.com).

### 3.2. Autosomal Recessive RP

Over 30 genes and loci (Table **4**) have been implicated in autosomal recessive RP (Fig. (**2**)), even if most of them are rare, causing 1% or fewer cases. However, for some of them (RPE65, PDE6A, PDE6B and RP25) percentages can be higher, up to 2-5% of cases, and will therefore be more thoroughly described.

#### Retinal Pigment Epithelium 65

Retinal pigment epithelium 65 (RPE65) is an isomerohydrolase expressed in retinal pigment epithelium and is critical for the regeneration of the visual pigment necessary for both rod and cone-mediated vision. More than 60 different mutations have been found in the RPE65 gene, accounting for approximately 2% of recessive RP cases and 16% of LCA patients. Several animal models, including the naturally occurring canine (Briard dog) and murine (*Rpe65^rd12^*) models and the genetically engineered *Rpe65-/-* knockout mouse, have been widely used for pathological, biochemical, genetic, structural, functional and therapeutic studies (reviewed in [22]). Because of all these relevant models, for RPE65 mutations the mechanism leading to the disease is fairly well understood. The vitamin A derivative 11-*cis*-retinal is the chromophore of rod and cone visual pigments. As previously described, absorption of light leads to an 11-*cis* to all-*trans* isomerization followed by dissociation of all-*trans*-retinal from the protein moiety (opsin) of the visual pigment holo-complex. Importantly, the crucial all-*trans* to 11-*cis*-retinoid isomerization reaction step takes place in the RPE and is catalyzed by RPE65. Rpe65-deficient mice, in fact, lack 11-*cis*-retinal and consequently no rhodopsin is detectable in their eyes. Instead, these animals show an over-accumulation of retinyl esters, retinoid intermediates of the visual cycle, in the RPE [23].

Results in pre-clinical studies have recently led to three encouraging gene therapy clinical trials in which patients affected by LCA were sub-retinal injected with recombinant adeno-associated viral vectors (rAAV) containing the human RPE65 cDNA [24-26]. Although the purpose of these initial studies was merely to test the safety of the gene transfer agent, all 3 groups did report modest (though significant) improvements in visual acuity. As a result of the groundbreaking positive reports, these three trials (NCT00516477, NCT00643747, NCT00481546, www.clinicaltrials.gov) are ongoing and expanding their patient population to examine treatment safety and efficacy further.

#### PDE6A, PDE6B, PDE6G

Among the autosomal recessive RP genes so far identified, many encode proteins important in the rod photoreceptor visual transduction cascade. Central among these is the heterotetrameric phosphodiesterase (PDE) 6 complex, consisting of α, β and two γ subunits. The PDE6 complex regulates the intracellular cGMP levels by hydrolyzing cGMP in response to light activation of G protein coupled receptors in cones and rods, thus making it an essential component of the visual phototransduction cascade [27]. In RP, mechanisms that initiate rod photoreceptor death are unclear; however, low levels of PDE6 activity are thought to lead to rod-cone degeneration. One hypothesis is that in PDE6 mutants an influx of calcium (Ca^2+^) causes rod apoptosis, due to lack of PDE activation. Pharmaceutical interventions to block excessive Ca^2+^ influx into rods have been attempted with variable success in mouse models (*Pde6b^rd1^/Pde6b^rd1^*mice) of the disease and Irish Setter dogs. Each subunit of the PDE6 complex is essential for photoreceptor function and maintenance, and, in fact, mutations within *PDE6A* (coding the phosphodiesterase 6A) and *PDE6B* (coding the phosphodiesterase 6B) genes are the second most common identifiable cause of arRP (after mutations in the *USH2A* gene). About 36,000 cases of simplex and familial RP worldwide are due to defects in *PDE6* complex, estimated to account for approximately 8% of all diagnosed arRP. In addition, mutations in the β-subunit of *PDE6B* can lead to either progressive retinal disease, such as RP, or stationary disease, such as congenital stationary night blindness (CSNB). Heterozygous carriers of *PDE6* mutations are at a risk of losing vision when excessively inhibiting PDE6 with drugs such as sildenafil, tadalafil, or vardenafil [28]. More recently, a mutation (c187+1G>T) in the PDE6G gene encoding the gamma subunit of the rod cGMP phosphodiesterase was reported, thus confirming the involvement of PDE6G in autosomal-recessive early-onset RP [29].

#### RP25

Other mutations, while rare elsewhere, can be a common cause of arRP in specific populations, such as the RP25 locus which was identified as the genetic cause of 10-20% of arRP cases in Spain [30].

#### Diagnosis of arRP

Tests available to screen mutations involved in arRP are so far well developed and able to detect up to 594 different mutations from several arRP-related genes including CERKL, CNGA1, CNGB1, MERTK, PDE6A, PDE6B, PNR, RDH12, RGR, RLBP1, SAG, TULP1, CRB, RPE65, USH2A, USH3A, LRAT, PROML1 and PBP3. In addition, many diagnostic laboratories offfer the possibility to have the entire coding region of the RPE65, PDE6 and USH2A genes sequenced.

### 3.3. X-Linked RP 

Around 10%-15% of RP patients have X-Linked RP (XLRP; Fig. (**3**)) and are characterized by a severe phenotype in the early stages of disease development. A milder phenotype can sometimes occur in female carriers, probably due to non-random or skewed inactivation of one X chromosome. In some patients, abnormal sperm development, impaired hearing and defects in the respiratory tract were observed, thus underlying the ciliary background of the disease (reviewed in [31]).

Six gene loci responsible for XLRP have been mapped on the X-chromosome (RP6, RP23, RP24, RP34, RP2 and RPGR – Table **5**), but only two of these have been identified so far: the retinitis pigmentosa GTPase regulator (*RPGR* or *RP3*) and the retinitis pigmentosa 2 protein (*RP2*). 

The *RPGR/RP3* gene product localizes in the outer segment of rod photoreceptors and is essential for their viability. Around 70% of XLRP patients have mutations in the *RPGR/RP3* gene. However, only 20% of the RP3 mutations occur within the exons coding for RPGR, while the ORF15 region turned out to be a mutational hotspot, with a mutation rate of 30-60% of all XLRP cases [32].

The predicted *RP2* gene product shows homology with human cofactor C, a protein involved in the ultimate step of beta-tubulin folding. Progressive retinal degeneration may therefore be due to the accumulation of incorrectly-folded photoreceptor or neuron-specific tubulin isoforms followed by progressive cell death. The gene responsible for RP2 was first cloned in 1998 and approximately 10-15% of XLRP patients have mutations in this gene. In contrast to *RPGR*, missense mutations and mutations leading to a truncated protein are distributed throughout the *RP2* sequence (reviewed in [31]).

Differently from other forms, in X-linked RP, the two genes RPGR and RP2 alone account for more than 80% of the clinical cases and are therefore better target candidates for small-molecule drug or gene therapy approaches. As for the genetic analysis, a screening test able to evaluate up to 184 mutations in RP2 and RPGR (excluding ORF15) genes is currently available (www.asperbio.com).

## 4. SYNDROMIC RETINITIS PIGMENTOSA

In the majority of cases, RP is an isolated disorder (simple or non-syndromic), but there are also syndromic forms. In addition, infrequently, RP can also be found associated with other systemic conditions such as abetalipoproteinemia and Refsum disease. For the purpose of this review paper, we will only describe the more frequent of these syndromes, namely Usher syndrome and Bardet-Biedl syndrome.

### 4.1. Usher Syndrome

Usher syndrome (USH) is an autosomal recessive disease characterized by the association of hearing loss, retinitis pigmentosa, and, in some cases, vestibular dysfunction. The syndrome is the most frequent cause of deaf-blindness, accounting for more than 50% of individuals who are both deaf and blind and affects between 1:12,000 – 1:30,000 people in different populations. The Usher cases may represent between 10-30% of all autosomal recessive RP cases (reviewed in [33]).

Clinically, USH is divided into three types. Usher type I (USH1) is the most severe form and is characterized by severe to profound congenital deafness, vestibular areflexia, and pre-pubertal onset of progressive RP. Type II (USH2) displays moderate to severe hearing loss, absence of vestibular dysfunction, and later onset of retinal degeneration. Type III (USH3) shows progressive postlingual hearing loss, variable onset of RP, and variable vestibular response.

To date, five USH1 genes have been identified: MYO7A (USH1B), CDH23 (USH1D), PCDH15 (USH1F), USH1C(USH1C), and USH1G(USH1G). Three genes are involved in USH2, namely, USH2A (USH2A), GPR98 (USH2C), and DFNB31 (USH2D). USH3 is rare except in certain populations (Finns and Ashkenazi Jews), and the gene responsible for this type is USH3A (Table **6**). 

Currently, there is no treatment available for USH. As for the diagnosis, an updated version of the Usher micro-array has recently been implemented by Asper Biotech (Tartu, Estonia) allowing to screen 612 mutations in the genes CDH23, MYO7A, PCDH15, Harmonin, SANS, USH2A, VLGR1, USH3A, and Whirlin.

### 4.2. Bardet-Biedl Syndrome

Bardet-Biedl syndrome (BBS) is characterized by rod-cone dystrophy (>90%), truncal obesity (72%), postaxial polydactyly, cognitive impairment, male hypogonadotrophic hypogonadism, complex female genitourinary malformations, and renal abnormalities. Renal disease is a major cause of morbidity and mortality. Birth weight is usually normal, but significant weight gain begins within the first year and becomes a lifelong issue for most individuals.

The visual prognosis for children with BBS is poor. Night blindness is usually evident by age seven to eight years and the mean age of legal blindness is 15.5 years. Atypical pigmentary retinal dystrophy with early macular involvement is the characteristic fundus abnormality in BBS [34, 35]. Visual acuity (central retinal function mediated by cones), dark adaptation, and peripheral visual fields (peripheral retina function mediated by rods) are all affected.

BBS is typically inherited in an autosomal recessive manner and affects 1 individual in 120,000 Caucasians. A higher incidence has been reported in isolated populations of Newfoundland (1 in 13000) [36] and among the Bedouins (ranging between 1:13500 and 1:6900 among the Bedouin population in the Jahra district) [37]. Fourteen genes are known to be associated with BBS: *BBS1* (mutated in 40% of BSS families), *BBS2* (mutated in 20% of BSS families), *ARL6/BBS3*, *BBS4* (mutated in 3-6% of BSS families), *BBS5* (mutated in 2% of BSS families), *MKKS/BBS6*, *BBS7*, *TTC8/BBS8*, *B1/BBS9*, *BBS10*, *TRIM32/BBS11, BBS12, MKS1/BBS13,* and *CEP290/BBS14* (from http://www.sph.uth.tmc.edu/retnet/). Molecular genetic testing is currently available on a clinical basis for all 14 known BBS-related genes. However, approximately 20% of persons with BBS do not have identifiable mutations in any of the 14 genes and therefore, it is possible that more BBS-related genes are yet to be identified.

## 5. FUTURE DIRECTIONS

A reasonable hope is that in the near future, molecular testing of newly diagnosed patients with RP will be a routine part of clinical practice and will uncover the underlying disease-causing mutations in at least 95% of cases. However, for this hope to come true, the following conditions will need to be met:

- Most of the genes causing RP must be identified. In spite of the large number of genes identified to date, the fraction of patients in which a mutation can be found by screening the known genes is often low;
- It should be possible to detect nearly all of the disease-causing mutations within these genes;
- Molecular testing should be inexpensive, reliable, fast and widely available;
- Clinicians must be able to understand, interpret and explain the molecular information.

Several strategies do exist and novel technologies are emerging that increase the chance to identify the causative mutation in patients. Array-based diagnostic tests have nowadays been established for several retinal diseases, including RP; but allow to detect only known mutations. Next generation sequencing techniques, that is, novel gene selection and targeting methods followed by massively-parallel, ultra-high-throughput sequencing, offer a rapid, efficient way to find disease-causing mutations in affected individuals and to discover new disease genes. These techniques have recently been applied to finding genes and mutations causing adRP in individuals/families in which conventional testing failed to detect mutations in known genes [38, 39].

However, an important challenge for all these genetic tests is the interpretation of the results obtained. Many of the DNA sequence variations lead to amino acid substitutions and it is difficult to predict the pathogenic effects for many of them. Functional assays would therefore be needed to characterize these sequence alterations, but, due to the extensive individuality of pathogenic and non-pathogenic sequence alterations, this approach would be difficult to realize in a routine diagnostic setting.

What we really need today are comprehensive databases integrating genetic and clinical data in order to perform detailed genotype-phenotype comparisons. This would allow to reveal important information not only to diagnose RP in a patient or family for genetic counseling, but it may also have a predictive value with respect to the likelihood of disease development and choices of therapy (nutrition supplements, drugs, gene replacement strategies, stem cell technologies, etc.) [40].

## Figures and Tables

**Fig. (1).**
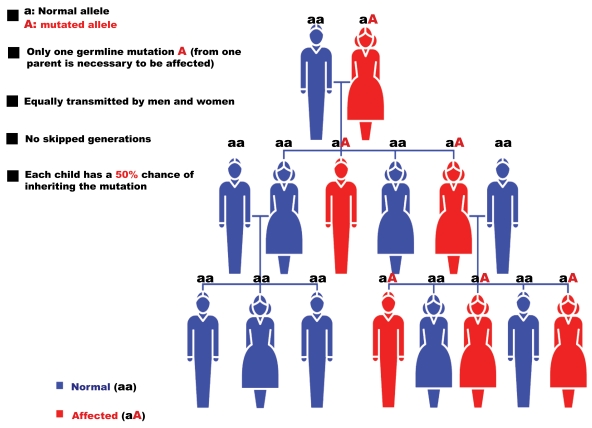
Pattern of autosomal dominant RP inheritance.

**Fig. (2).**
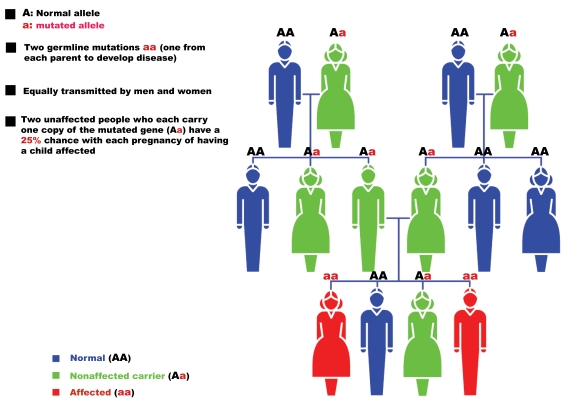
Pattern of autosomal recessive RP inheritance.

**Fig. (3).**
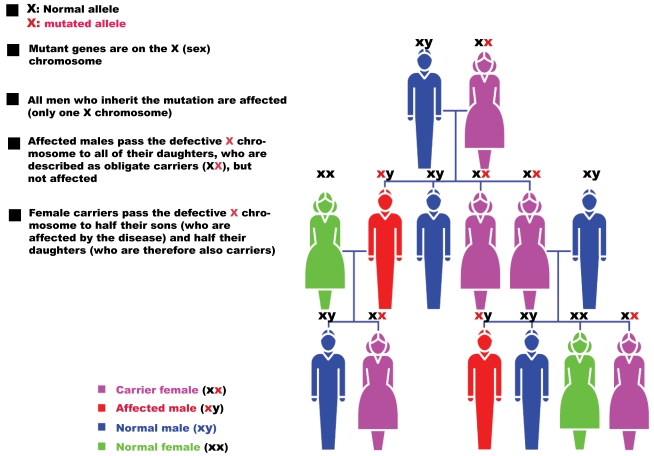
Pattern of X-linked RP inheritance.

**Fig. (4).**
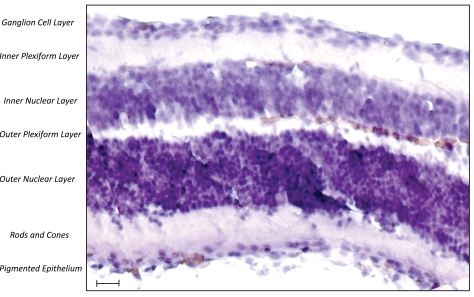
Haematoxylin-stained section of the human retina (scale bar = 100µm).

**Table 1.** Summary of Genes Associated with RP (Adapted from http://www.sph.uth.tmc.edu/retnet/)

Disease	Total No. of Genes and Loci	No. of Identified Genes
Leber congenital amaurosis, autosomal dominant	1	1
Leber congenital amaurosis, autosomal recessive	9	8
Retinitis pigmentosa, autosomal dominant	17	17
Retinitis pigmentosa, autosomal recessive	24	21
Retinitis pigmentosa, X-linked	6	2
Usher syndrome, autosomal recessive	11	9
Bardet-Biedl syndrome, autosomal recessive	12	12

**Table 2.** Estimated Percentages of RP Types

Category	Type	%
Non-syndromic RP	Autosomal dominant RP	20-25
	Autosomal recessive RP	15-20
	X-Linked RP	10-15
	Leber congenital amaurosis	4
	Digenic RP	Very rare
Syndromic and systemic RP	Usher syndrome	10
	Bardet-Biedl syndrome	5

Abbreviations: RP = Retinitis Pigmentosa.

**Table 3.** Genes, Proteins and their Functions in Autosomal Dominant RP (Adapted from [6, 9] and http://www.sph.uth.tmc.edu/retnet/)

Gene	Protein	% of all cases	Function	Other diseases/phenotypes
BEST1	Bestrophin-1		Anion channel	Recessive RP
CA4	Carbonic anhydrase IV	Arg14Trp found in 4% of Swedish controls	Zn-containing enzyme that catalyzes hydration of carbon dioxide	None
CRX	Cone-rod homeobox protein	1,0	Transcription factor	Recessive and dominant LCA, dominant CORD
FSCN2	Fascin homolog 2, actin-bundling protein		Cellular structure	None
GUCA1B	Guanylate cyclase activator 1B		Phototransduction	Dominant MD
IMPDH1	Inosine 5’-monophosphate dehydrogenase 1	2,5	Regulation of cell growth	Dominant LCA
KLHL7	Kelch-like 7		Ubiquitin-proteasome protein degradation	None
NR2E3	Nuclear receptor subfamily 2, group E, member 3		Transcription factor	Recessive RP; Recessive enhanced S-cone syndrome
NRL	Neural retina leucine zipper protein		Tissue development and maintenance	Autosomal recessive RP
PRPF3	PRP3 pre-mRNA processing factor 3 homolog (S cerevisiae)	1,0	Splicing	None
PRPF8	PRP8 pre-mRNA processing factor 8 homolog (S cerevisiae)	3,0	Splicing	None
PRPF31	PRP31 pre-mRNA processing factor 31 homolog (S cerevisiae)	8,0	Splicing	None
PRPH2 (RDS)	Peripherin 2	5-9,5	Photoreceptor outer segment structure	Digenic forms with ROM1
RDH12	Retinol dehydrogenase 12		Phototransduction	Recessive LCA
RHO	Rhodopsin	26,5	Phototransduction	Recessive RP, dominant CSNB
ROM1	Retinal outer segment membrane protein 1		Cellular structure	Digenic RP with PRPH2 (RDS)
RP1	Retinitis pigmentosa 1	3,5	Tissue development and maintenance	Recessive RP
RP9	RP-9		Splicing	None
SEMA4A	Sema domain, immunoglobulin domain (Ig), transmembrane domain (TM) and short cytoplasmic domain (semiphorin) 4A		Tissue development and maintenance	Dominant CORD
SNRNP200	Small nuclear ribonucleoprotein 200kDa (U5)		Splicing	None
TOPORS	Topoisomerase I binding, arginine/serine-rich		localized in the basal body of connecting cilia in photoreceptors	None

Abbreviations: RP = Retinitis Pigmentosa; LCA = Leber congenital amaurosis; CSNB = congenital stationary night blindness; MD = macular dystrophy; CORD = cone-rod dystrophy.

**Table 4.** Genes, Proteins and their Functions in Autosomal Recessive RP (Adapted from [6, 9] and http://www.sph.uth.tmc.edu/retnet/)

Gene	Protein	% of all cases	Function	Other diseases/phenotypes
RP22	Unknown		Unknown	None
RP29	Unknown		Unknown	None
RP32	Unknown		Unknown	None
ABCA4	ATP-binding cassette, subfamily A (ABC1), member 4	2,9	Retinal metabolism	Recessive MD, recessive CORD
BEST1	Bestrophin-1		Anion channel	Dominant RP
C2ORF71	Chromosome 2 open reading frame 71		Unknown	
CERKL	Ceramide kinase-like protein		Tissue development and maintenance	None
CNGA1	Cyclic nucleotide gated channel alpha1	2,3	Phototransduction	None
CNGB1	Cyclic nucleotide gated channel beta1		Phototransduction	None
CRB1	Crumbs homolog 1	6,5	Tissue development and maintenance	Recessive LCA
EYS (RP25)	Eyes shut homolog		Protein of the extracellular matrix	-
FAM161A	Family with sequence similarity 161 member A		Unknown, localized in the photoreceptors	-
IDH3B	NAD(+)-specific isocitrate dehydrogenase 3 beta		Involved in Krebs cycle	-
IMPG2	Interphotoreceptor matrix proteoglycan 2		Component of the retinal intercellular matrix	-
LRAT	Lecithin retinol acyltransferase		Retinal metabolism	Recessive LCA
MERTK	C-mer proto-oncogene tyrosine kinase		Transmembrane protein	None
NR2E3	Nuclear receptor subfamily 2, group E, member 3		Transcription factor	Dominant RP; Recessive enhanced S-cone syndrome
NRL	Neural retina leucine zipper protein		Tissue development and maintenance	Dominant RP
PDE6A	Phosphodiesterase 6A, cGMP-specific, rod alpha	4,0	Phototransduction	None
PDE6B	Phosphodiesterase 6B, cGMP-specific, rod beta	4,0	Phototransduction	Dominant CSNB
PDE6G	Phosphodiesterase 6G, cGMP-specific, rod gamma		Phototransduction	
PRCD	Progressive rod-cone degeneration		Unknown function	-
PROM1	Prominin 1		Cellular structure	Recessive RP with macular degeneration
RBP3	Retinol binding protein 3		Retinal metabolism	-
RGR	Retinal G protein-coupled receptor		Retinal metabolism	Dominant choroidal sclerosis
RHO	Rhodopsin		Phototransduction	Dominant RP
RLBP1	Retinaldehyde-binding protein 1		Retinal metabolism	Recessive Bothnia dystrophy
RP1	RP-1 protein		Tissue development and maintenance	Dominant RP
RPE65	Retinal pigment epithelium-specific 65kDa protein	2,0	Retinal metabolism	Recessive LCA
SAG	S-antigen; retina and pineal gland (arrestin)		Phototransduction	Recessive Oguchi disease
SPATA7	Spermatogenesis associated protein 7		Unknown	
TTC8	Tetratricopeptide repeat domain 8		Cellular structure	Recessive Bardet-Biedl syndrome
TULP1	Tubby-like protein 1		Tissue development and maintenance	Recessive LCA
USH2A	Usher syndrome 2a	10,0	Cellular structure	Recessive Usher syndrome
ZNF513	Zinc finger protein 513		Expression factor	

Abbreviations: RP = Retinitis Pigmentosa; LCA = Leber congenital amaurosis; CSNB = congenital stationary night blindness; MD = macular dystrophy; CORD = cone-rod dystrophy; cGMP = cyclic guanosine monophosphate.

**Table 5.** Genes, Proteins and their Functions in X-Linked RP (Adapted from [6, 9] and http://www.sph.uth.tmc.edu/retnet/)

Gene	Protein	% of all cases	Function	Other diseases/phenotypes
RP2	Retinitis pigmentosa 2 protein	15,1	Human cofactor C is involved in beta-tubulin folding	None
RP6	Unknown		Unknown	None
RP23	Unknown		Unknown	None
RP24	Unknown		Unknown	None
RP34	Unknown		Tissue development and maintenance	None
RPGR	Retinitis pigmentosa GTPase regulator	74,2	Intraflagellar transport	X-linked COD, X-linked CSNB

Abbreviations: RP = Retinitis Pigmentosa; COD = cone dystrophy; CSNB = congenital stationary night blindness; GTPase = guanosine triphosphatase.

**Table 6.** Genes and Proteins (Including Function) Involved in Usher Syndrome (Adapted from [33])

Locus	Gene/protein	Function
*USH1B/DFNB2/DFNA1*	*MYO7A*/myosin VIIA	IE and R: transport
*USH1C/DFNB18*	*USH1C*/harmonin	IE and R: scaffolding
*USH1D/DFNB12*	*CDH23*/cadherin23	IE: tip link formation; R: periciliary maintenance
*USH1E*	-/-	Unknown
*USH1F/DFNB23*	*PDCH15*/protocadherin 15	IE: tip link formation; R: periciliary maintenance
*USH1G*	*USH1G*/SANS	IE and R: scaffolding and protein trafficking
*USH1H*	-/-	Unknown
*USH2A/RP*	*USH2A*/usherin	IE: ankle links formation and cochlear development; R: periciliary maintenance
*USH2C*	*GPR98*/VLGR1	IE: ankle links formation and cochlear development; R: periciliary maintenance
*USH2D/DFNB31*	*DNFB3*/whirlin	IE: scaffolding and cochlear development; R: scaffolding
*USH3A*	*USH3A*/clarin-1	IE and R: probable role in synapsis transport
*USH3B*	-/-	Unknown

Abbreviations: USH = Usher syndrome; DFNB = autosomal recessive deafness; DFNA = autosomal dominant deafness; RP = retinitis pigmentosa; IE = inner ear; R = retina.
